# Insistent individualisation and eroding egalitarianism: Dominant discourses of equality in Swedish healthcare policy

**DOI:** 10.1186/s12913-026-15824-0

**Published:** 2026-10-09

**Authors:** Marcus Lauri, Anna-Karin Hurtig, Per E. Gustafsson, Ida Linander

**Affiliations:** 1 Department of Education, Psychology and Social Work, Mid Sweden University, Östersund, Sweden; 2 Department of Epidemiology and Global Health, Umeå University, Umeå, Sweden

**Keywords:** Equality, Swedish healthcare policy, Healthcare reforms, Discourse analysis, Geographical equality, Individual agency, Patient responsibility, Liberal equality, Social determinants of health, Healthcare inequalities

## Abstract

**Background:**

Equality is a core objective that is frequently invoked in Swedish healthcare policy, yet its meaning is rarely examined in depth. This study analyses how equality is articulated in two contemporary healthcare reforms. The aim is to map the extent to which discussions on equality are present and how it is assumed that equality is to be achieved, analyse the meanings of equality that are produced, and discuss and problematise possible implications.

**Methods:**

The study applies discourse theory and conceptual discussions on equality, in the analysis of two major Swedish governmental reform reports: one on highly specialised care (SOU 2015:98) and the other on primary healthcare (SOU 2020:19).

**Results:**

Three meanings of equality were identified, of which the first two are more commonly articulated. Firstly, equality is articulated as sameness and uniformity, particularly in relation to geographical access, coordination across actors and locations, and the standardisation of care through knowledge production and exchange. Secondly, equality is framed as individual agency and patient responsibility, emphasising patients as co-creators of care through mechanisms such as individual care plans and patient contracts. To a lesser extent, equality is also articulated as responsiveness to healthcare needs. The two dominant meanings reflect liberal and individualised notions of equality that prioritise uniform access and the removal of difference, alongside an emphasis on individual responsibility.

**Conclusions:**

The analysis suggests that these dominant articulations marginalise more egalitarian or distributive understandings of equality that would take into account social determinants of health and unequal structural conditions. While equality occupies a central position in Swedish healthcare policy, its meaning is increasingly shaped by liberal values rather than commitments to social justice or redistribution. This has important implications for how healthcare reforms are designed and for the capacity of policy to address healthcare inequalities in practice.

## Introduction

The Swedish welfare state has been characterised by public ownership and the state-led provision of welfare services as an outspoken strategy to ensure equality [1], yet with a degree of professional autonomy [2]. Values of fairness, social solidarity, and universal service provision have long been central to Swedish healthcare policy and discourse, reflecting the broader Nordic care regime’s defining commitment to equality [3, 4]. Equality is underscored in the national policy for Sweden’s publicly funded healthcare system, stipulated in the preamble clause of the Health and Medical Services Act, to “provide care on equal terms” [5]. An overarching goal of Swedish government public health policy is to promote “a good and equal health in the entire population” with the aim of achieving ”more equal living conditions and thereby the possibilities for equal health” [6].

While these formulations reveal an outspoken commitment to various aspects of healthcare equality, studies show that Swedish healthcare has undergone dramatic changes in terms of its organisation and governing since the early 1990s [4, 7], which may affect healthcare equality ambitions [1, 8, 9].

One such change is marketisation, a process by which market ideals and mechanisms are introduced into welfare services that are traditionally provided or heavily regulated by the state, intended to spur competition between providers and increase cost efficiency [10, 11]. Studying a policy process in elder-care, Andersson and Kvist [12] found that equality was represented as “equal competition” between care providers or as female entrepreneurship. Studies on marketisation discourse suggest that its dominance may hinder attempts by governments and policymakers to consider alternatives [13]. A second change is new public management (NPM), a private-sector-inspired managerial governing approach illustrated in knowledge governing, performance measurement, and professional accountability [14]. Analyses of Swedish healthcare governance reports show a discourse of knowledge-based governance, where compliance with clinical guidelines for treatments and therapies is associated with equality [2].

A third change in healthcare organisation and governing is patient-centred care, where consumer choice and individualised services are positioned as central ideals [4, 8]. This has been shown to normalise patient choice [15] and represent elders’ non-choice of care provider as an anomaly [16]. Others have shown how a freedom-of-choice discourse shapes people into customers and positions those who lack the knowledge and/or ability to make choices as weak patients [17]. Health inequalities are represented as a lack of patient knowledge [18], and “freedom of choice” produces the responsibilisation of elders [19, 20] and entitlement for patients [21]. Meagher and Szebehely [1] suggest that the dominant ideals of choice and service diversity can alter egalitarian ideals.

Studies have suggested that healthcare policies discursively produce subjectification through notions of gender, race, culture, class, and chronical illness, thereby shaping the understanding of inequality as being a result of individual rather that structural factors [18, 22, 23; see also 24, 25]. According to Lynch [26], such representations may undermine policies that tackle the causes of health inequalities. We argue, in line with Blanchet Garneau et al. [27], that, in order for healthcare equality ambitions to be fruitful and progressive, it is essential to critically engage with the discursive frameworks that challenge equality and perpetuate inequality. This entails empirically analysing what equality in healthcare is taken to mean in a particular context.

An arena where discourses can be reproduced and challenged is government policies. Policy provides a litmus test for which discourses are given a central position and what meanings are given to central concepts, including equality, in a particular context such as healthcare. How such discourses are formulated provides guidance for future reforms; hence, policies do not merely reflect but also actively shape how healthcare is organised and delivered. Furthermore, because government policy constitutes an authoritative text in the sense that it is stated from a position of power, such policy exerts influence public opinion and, more broadly, healthcare equality discourse and notions of equality in general. Consequently, we do not regard policy as a neutral response to already-existing problems, but as highly influential in determining what is talked about, how something comes to be understood, and what other possible meanings are thereby silenced [28]. Which meaning of equality becomes dominant and which meaning(s) are silenced will affect how inequality is addressed, both in a healthcare context and in a general socio-political context.

While some studies have analysed equality in healthcare settings, to our knowledge, no one has studied the discourse of equality in Swedish healthcare policy, i.e., what meaning(s) of equality is/are articulated (however, see [29] for a study on discursive constructions of patients). This study therefore fills a knowledge gap, and provides an important foundation for further discussions on progressive healthcare and the political influence of government through the shaping of discourse, i.e. how something is talked about and understood.

For these reasons, *the aim* of this study is to analyse equality discourse in two recent yet distinct healthcare policies, one on highly specialised care and the other on primary healthcare. We want to map out the extent to which discussions on equality are present and how it is assumed that equality will be achieved, analyse what meanings of equality are produced, and discuss and problematise the possible implications of contemporary discourse(s) of equality in Swedish healthcare policy.

### Equality in Swedish healthcare policy: a conceptual framework

A discussion on the concept of equality in a Swedish context is called for. In the Swedish language, the word equality (*jämlikhet*) has been used at least from the 1700s [30], and can express multiple meanings [31]. A distinction between equality and equity is sometimes made in the international literature on healthcare, where equality typically refers to uniform access or treatment, and equity emphasises fairness based on differing needs and circumstances [32]. Furthermore, *horizontal* equity means that people with equivalent needs receive equivalent care, while *vertical* equity means that people with different needs are treated differently, in proportion to their needs [33]. However, the distinction between equality and equity does not exist in the Swedish language, therefore both equity and equality can be included in the term *jämlikhet*, from here on translated as “equality”.

Although no studies have examined how equality is articulated in Swedish healthcare policy, Burström [34 pp. 410–411] describes two sides of the public debate on healthcare equality, a marketisation side that seeks “to maximize personal freedom and choice” and the egalitarian side, which seeks to “reduce disparities in human circumstances and opportunities”. We have also drawn upon Wedin’s [35] conceptual distinction between different articulations of equality. Wedin argues that, historically, equality has been framed on the one hand as a matter of equality before the law (i.e. formal equality) and, on the other, as a matter of equal distribution of resources (what he calls distributive equality), similar to Burström’s second egalitarian aspect. Wedin also identifies a third, more recent, form in Swedish education policy, which he calls an “imaginary equality”, which focuses on the individual with the aim of hearing and seeing each individual “here and now” in accordance with her own self-understanding and without contextualised aspects. Together with the insights from previous research on marketisation, NPM, and patient-centred care, this discussion will serve as a theoretical and conceptual framework for our analysis.

## Method and material

We have conducted a policy analysis of two governmental reports on two ongoing healthcare reforms in Sweden; one from 2015 on highly specialised care and one from 2020 on primary healthcare. The goal of the highly specialised care reform is to centralise services for more rare conditions and enable higher volumes of treatment for certain complex medical services to improve quality, efficiency, and patient outcomes. Highly specialised care is limited to a maximum of five units per treatment area (examples being organ transplants, severe psychiatric illness, etc.), with decisions made by the national authorities. The primary healthcare reform, “Good and local care” [God och nära vård], aims to re-establish primary care as the frontline of care and emphasises person-centred care, improved local availability, continuity, and collaboration between municipalities and regions [36]. Together, these inquiries represent critical policy moments in contemporary Swedish healthcare reform and illustrate different approaches to decentralisation and specialist care/primary care, providing various contemporary empirical examples through which to analyse shifting discourses on equality in Swedish healthcare policy. The material is analysed using discourse theory, drawing on the works of Howarth, and Laclau and Mouffe (see below).

### Study setting

The Swedish healthcare system is primarily tax-funded, and responsibility for healthcare provision lies mainly with the 21 regional authorities, which organise and finance services, while the national government sets overall policy goals and legislation. Municipalities are responsible for disability care, some rehabilitation, home-based care, elder care, and school health services.

The two policies and the analysis of healthcare equity can be understood in the context of broader transformations in the Swedish healthcare system over the past 20–30 years (for a historical overview see [4]). Since the 1990s, the system has undergone significant changes and is increasingly shaped by market-oriented reforms. During the 1980s, 1990s, and 2000s, reforms focused on introducing competition, increasing freedom of choice, privatisation, and performance-based reimbursement systems. In more recent years, policy efforts have shifted towards enhancing patient-centredness, increasing accessibility, promoting digitalisation, reducing waiting times, and boosting efficiency and quality. Several of these reforms have also emphasised the strengthening of primary healthcare [4, 37, 38, 39]. This context provides a particularly relevant setting for examining policy discourses, because Sweden is often portrayed internationally as having a model of universal healthcare, while at the same time undergoing structural and ideological shifts that may reshape how equality is articulated and pursued in practice.

### Analytical approach

We start from the assumption that words have no original or essential meaning, that there is no factual “correlation between words and things, or an inherent characteristic of texts, objects or practices” [40, p. 10]. Instead, meaning is actively and constantly (re)produced, and is *relational*, an effect of the “differences between terms” [40, p. 10]. This means that language is not a reflection of an already-existing reality; rather, the understanding of reality is a constitutive effect of language. Consequently, when we speak or write, we use already-existing notions of words – their temporarily fixed meanings – but every “speech act” entails the potential (re)creation of the meaning of a word, which explains how the meanings of words can vary depending on context and shift over time.

In our analysis of equality, we make use of selected analytical concepts from discourse theory to untangle meaning-making processes in healthcare policy: (dominant) discourse, articulation, chains of equivalence, floating signifiers, subjectification, and exclusion, which are explained below.

*Discourse* refers to the ways in which language is used in texts and contexts to construct social realities and how it “constitutes and organizes social relations” [41, p. 96] and discourses are “historically specific systems of meaning which form the identities of subjects and objects” [40, p. 9]. This means that how something is talked about will shape its understanding and practice. Tracing *dominant discourses* in healthcare policy helps illustrate authoritative meanings, which can shed light on how such discourses frame and limit the understanding(s) of equality. We examine *articulations*, the practice of producing meaning in language, through relating one word with another, often through difference. Articulation also creates meaning by connecting several words together by association, in *chains of equivalence*, and we use this analytical concept to identify how the articulation of equality creates and dislocates meaning through its association with other words [41, p. 93]. Tracing *floating signifiers* is a way of illustrating how some words can be filled with various meanings depending on context.

As explained above, an important aspect of articulation – the practice of meaning-making in language – is that of difference and exclusion: “a discourse always requires a discursive ‘outside’ to constitute itself” [40, p. 103]. The meaning of words is achieved by the exclusion of other meanings and therefore a word is defined by its constitutive outside, by what it is not. Foucault highlights the “dividing practices” of discourse, where the “subject is either divided inside himself or divided from others” [42, pp. 777–778]. Paying attention to how discourse produces certain kinds of *subjectification* illustrates how these shape a particular understanding of (in)equality, and using the analytical term *exclusion* allows us to study what has been “pushed out” of the discourse, i.e. what other meanings or possibilities are silenced.

### Empirical material

The empirical material in this article consists of two Swedish Government Official Reports (SOU). The first one is the highly specialised care inquiry (*Träning ger färdighet: Koncentrera vården för patientens bästa*, SOU 2015:98 [43]), which comprises 268 pages and was initiated by a centre-right government coalition. The second is the primary healthcare inquiry (*En god och nära vård: En reform för ett hållbart hälso- och sjukvårdssystem*, SOU 2020:19, the main report of the inquiry (*huvudbetänkande*) [36]), comprising 492 pages and initiated by a social-democratic government. Both inquiries propose reforms aimed at restructuring healthcare delivery and improving or developing healthcare equality but have different scopes and logics. The 2015 inquiry on highly specialised care (henceforth the “HSC report”) sought to concentrate advanced medical services for more rare conditions in fewer hospitals in order to ensure quality, patient safety, equality, and cost-effectiveness. In contrast, the 2020 inquiry on primary healthcare (henceforth the “PHC report”), emphasised the strengthening of frontline services, continuity, and patient-centredness, positioning primary care as the foundation of healthcare provision.

### The analytical process

We analyse the meanings that equality is given in policy, through practices of articulation, i.e. how it is related or positioned to other words, through antagonistic relations of opposition or its association with other meanings in chains of equivalence. Of particular interest is the meaning that equality is given in this particular context, how it is articulated across the two policies, and what the implications are in terms of the exclusion of other possible meanings. In other words, how healthcare policy interpellates, creates, and mobilises subjects, and how it produces social meanings, social divisions, and identities [40, p. 137].

More concretely, we have conducted in-depth analyses of paragraphs or sections where the word *jämlik* [equal] and derivations thereof appears. This means that we have captured *ojämlik* [unequal], *jämlik* [equal], *ojämlikhet* [inequality] and *jämlikhet* [equality]. In addition, we have searched the reports for other terms with potentially similar meanings, such as *likvärdig* [equivalent], *rättvis* [fair] and dealing with non-discrimination, *diskriminering*, to discern if they were given a central place in the policy texts. This supplementary search did not yield any substantial findings, indicating that *jämlik* (and its variants) is the primary term used to articulate issues of equality in the policy texts.

The analytical process involved several iterative steps. Firstly, all parts containing the word *jämlik* (including its variations) were identified and extracted. These excerpts were then subjected to an initial, inductive round of coding, allowing the identification of key ideas and patterns. In the next stage, the codes were grouped into preliminary broader categories/domains, which provided an initial structure for the analysis. Writing the analysis itself became an integral part of the analytical process: as we developed each section, we revisited and revised the categorisation/structure. Throughout this process, we read the material in dialogue with the aforementioned previous research and the conceptual framework on equality, which helped us to refine the analytical lens and situate the findings within a broader theoretical and empirical context.

## Findings

Equality was a central explicit objective of both reforms. In both reports, equality is something to be developed, enhanced, and established (*omställning till*). The word *jämlik*/equality (capturing all the four versions: *ojämlik*, *jämlik*, *ojämlikhet*, and *jämlikhet*) appears 25 times in the HSC report and 116 times in the PHC report, with the most common expression being “equal care”.

Before we delve into untangling the different meanings given to the word equality (see also Table 1), it is noteworthy to mention that equality is also used on occasions where there is no articulation, where no meaning is given to it. Such uses appear in both policies, but most frequently in the PHC report. For example, equality is part of the listing of traits of an ideal healthcare, including the title of the instruction given to the inquiry “Coordinated development of a modern, equal, accessible, and efficient healthcare system, with a focus on primary care”. This particular enumeration, “modern, equal, accessible, and effective healthcare” is in fact listed 16 times, with no articulation ever made of what equal healthcare means. In addition to this, the words equal and equality are used in several other instances in both reports, without any articulation of its meaning (see for example pp. 55, 345, 418, 458, 478 of the PHC report [36]). In the concluding discussion, below, we return to what this “non-meaning”, together with the different meanings that we now analyse in-depth, implies for the discourse of equality.

**Table 1:** Overview of findings

Sections	Sub-sections
1. Equality as sameness and uniformity	1a. Equality as lack of geographical variation in the provision of care 1b. Collaboration, coordination, and centralised decision-making for equality 1c. Equality as knowledge production and information exchange for sameness
2. Equality as increased agency and responsibility for individual patients	
3. Equality as being responsive to the diverse needs of different groups	

### Equality as sameness and uniformity

Prevalent articulations of equality in both reports include lack of geographical variation (1a), achieving equality through collaboration and coordination between locations and providers, with centralised decision-making (1b), and knowledge production and information exchange (1c), each contributing to a recurring discourse of equality as sameness and uniformity.

#### a) Equality as lack of geographical variation in the provision of care

A frequent articulation of equality concerns the absence of geographical differences, as *healthcare being the same* in different locations or as *availability being the same* everywhere in Sweden. When it comes to healthcare being the same, the PHC report states:

For increased national equivalence in primary care, with reduced regional and local differences, and to ensure good and equal care throughout the country, we put forward in our second interim report proposals for the regulation of what is to constitute the basic mandate of primary care [36, p. 254] 1

Availability being the same is articulated in the HSC report as:

Equal access to highly specialised care must be ensured. The patient’s place of residence, age, level of education, ethnicity, etc., should not determine access to the best care. If the treatment is provided at a few locations in the country, access to highly specialised care should be the same regardless of whether the patient lives near or far from the specialist care [43, p. 44]

Taken together, healthcare equality is articulated in both policies as sameness: “equivalence”, “reduced regional and local difference”, and where aspects such as place of residence or distance from healthcare should not determine access. It constructs healthcare equality through the exclusion of difference. This aligns with an understanding of equality as meaning that all individuals should be treated the same; in this case, regardless of their geographical location. The PHC report also articulates geographical healthcare equality as a societal obligation:

Part of the goal for health and medical care regarding care on equal terms means that health and medical care should be equal […] Society has an obligation, within the framework of available resources and knowledge, to ensure that care is offered to anyone in need of care, regardless of where they live. In other words, care resources should be fairly distributed across geographical areas [36, p. 276]

Offering care to anyone in need, independent of where they live, can be read as an articulation of health equality as formal equality (see [35]), an individual formal right, framed as a responsibility of society, and with resources fairly distributed geographically.

Another way of achieving equality in terms of the same local availability in the PHC reform is to facilitate the establishment and subsidising of small (private) healthcare actors in rural areas:

This nationally designed system aims to create long-term stability and better conditions for equal care across the country, and enables the establishment of smaller health and medical care providers in sparsely populated areas. The regions [regional authorities] are given good planning conditions, and by proposing that the state co-finance the care, incentives are created for establishments in areas where the provision of care is limited [36, pp. 307–308]

Locally available, and thus equal, healthcare is constructed as an outcome, negotiated between different levels (national/state/regional) and actors (public/private), managed through planning, financial incentives, and legislative structures. In the majority of the geographical articulations of equality, the focus lies on shortcomings in sparsely populated areas. One exception can be found in the PHC report, where the advantages of metropolitan areas are highlighted:

Healthcare today is unevenly distributed across the country, with a disproportionately high availability in metropolitan areas and low availability in sparsely populated areas, where there are substantial unmet needs. This proposal provides the regions with new methods and state funding to meet healthcare needs in rural areas. This enables more equal care across the entire country [36, p. 332]

The majority of the geographical articulations of equality in the material are limited to differences between regions and between urban and rural spaces, rather than between residential areas *within* cities, which may (also) be linked to socioeconomic conditions. This focus thus silences differences in access (understood as the whole process from identifying need to utilising services, see [44]) between affluent and marginalised settings

In the HSC report, geographical healthcare equality is articulated in a somewhat different way:

Against this background, the Government concludes that there is a need to review how highly specialised care can be further concentrated compared to today, with the aim of achieving improved care outcomes and thus more equal care, as well as attaining a more efficient use of health and medical care resources [43, p. 38]

Here, equality is articulated through a chain of equivalence in which the centralisation and concentration in provision of highly specialised services are expected to produce improved outcomes and thus more equal access to high-quality care. Equality is thus articulated as having access to the same quality of care, while the articulations seen in the PHC reform – proximity, local availability, or geographical coverage – are absent.

Hence, in articulating geographical equality, issues of decentralised and centralised provision of care become important. The two reports may at first glance be seen as articulating geographical (in)equality differently, with the HSC reform aiming to centralise care provision for more rare conditions and the PHC reform to decentralise services by facilitating the establishment or development of healthcare practices in rural areas. However, both articulate equality as a matter of geographical sameness. This articulation positions decentralised decision-making, with its potential for local adaptation and autonomy, and an understanding that some areas might need more resources than others, as a threat to equality.

#### b) Collaboration, coordination, and centralised decision-making for equality

In this second part of geographical sameness, we focus on how collaboration (Sw: *samverkan*) between levels and providers, coordination (Sw: *samordning*) and centralised decision-making are framed as something that will lead to equality. A key aspect is that collaboration is framed as a response to decentralised or fragmented decision-making:

In balancing the national values that patients should have more equal access to highly specialised care of good quality, better health, and more efficient use of resources, against the county councils’ interest in ensuring that municipal self-government is not restricted, it is clear to the inquiry that the national values concerning good and equal care carry greater weight [43, p. 223]

This also brings in factors such as coordination across the country – when mobility increases for both patients and staff, the need for coordination also increases in what may sometimes be 20 [regions] or 290 [municipalities] different ways of providing a service. This becomes a matter of […] equal care [36, p. 103]

In the quotes above from both reports, regional and municipal autonomy is pitted against national interests, and coordination across the country, hence articulating equality as geographical sameness. Although the meaning of equality is articulated similarly, the underlying rationale differs between the reports:

The inquiry into highly specialised care has the task of proposing how highly specialised care can be developed so that patients have access to better and more equal health and medical care. We propose that this can be made possible through increased national governance [43, p. 15]

In this quote from the HSC report, the logic seems to be *centralised coordination for centralised services* (see also p. 153 in the HSC report), where equality is assumed to be achieved through national-level coordination and concentration, and coordination mainly concerns the national level and between regions. In the PHC report, there is also a focus on the regional level and between municipalities and the counties as different providers:

We consider that the requirement to establish a joint plan for the development of health and medical care at the shared level of primary care in the county is an appropriate way to achieve increased coordination. […] However, this does not happen, as each responsible provider largely plans only from the perspective of their own organisation. This results in missed potential coordination gains and thus lost opportunities for efficiency, as well as creating poorer conditions for providing coherent, good, and equal care to the county’s residents [36, p. 163].

Taken together with the previous section (1a), where the PHC report focuses on locally available services, this report displays a somewhat different logic of centralisation compared to the HSC report: *centralised coordination (decision-making) for decentralised services*. National or regional steering is presented in the PHC report as necessary in order to align different providers, overcome fragmentation, and create conditions for locally distributed provision. Equality is therefore articulated as the outcome of centrally coordinated but decentralised provided services. In both reports, however, fragmentation and the different perspectives of different providers challenge healthcare equality and frame coordination as necessary. Both reports also link coordination to equality through sameness, as exemplified above, and below in the primary-care policy:

Clearer regulation of collaboration at the provider level contributes to more equal health and medical care across the country, as the requirements for collaboration will be applied more consistently between different principals, forms of operation, and providers [36, p. 196]

In this quote, equality is explicitly tied to uniformity; “applied more consistently”, with regulation and collaboration being expected to produce standardisation across diverse providers. Equality is thus constructed less as having to do with access to care, health outcomes, or as a matter of distributive equality [35] and more as the achievement of sameness in practice through coordination, national management, uniformity, and standardisation.

#### c) Equality as knowledge production and information exchange for sameness

Articulations in both reports establish a connection between knowledge production and equality. This is done in different ways, one of which links education to equality:

To ensure good and equal care across the whole country […] we assessed that it […] should be included in the core mission of primary care […] to contribute to the training of students and professionals in the professions present in primary care [36, p. 254]

The quote above articulates that including primary caregivers in education will produce “equal care across the whole country”, where “the whole country” alludes to handling possible geographical differences. In the HSC report, it is stated that:

The need to exchange information will increase as patients increasingly move across county council borders and between public and private care. Effective and appropriate information management is therefore one of the basic prerequisites for equal care and services of high quality across the whole country [43, p. 60]

Here, the exchange of information between caregivers is represented as a prerequisite for equality and this too is related to possible geographical differences. These two quotes articulate that increasing caregivers’ knowledge can help them to provide equal care in the sense of the same care, across the whole country. This is similar to the previous sections (1a and 1b), albeit with a different logic.

A similar articulation, but as a more outspoken *means to govern* healthcare, appears in the following two excerpts:

Small county councils have difficulty gathering the critical mass required, among other things, to make effective investments in healthcare in order to conduct research and provide care of high quality […] Overall, the Responsibility Committee assessed that larger regional municipalities would create better conditions for knowledge-driven health and medical care and thereby improve equality in care [43, p. 58]

Another central component of this concerns access to up-to-date and relevant knowledge for equal care. Here, the inquiry particularly wishes to highlight the need to prioritise the development of support adapted to the circumstances of both municipally provided and region-financed primary care [36, p. 125]

Under the heading “Governing with knowledge” (excerpt below), it is further stated that the goal of the expert groups is to

Gradually […] replace parts of, but also complement, the county councils’ work with regional and local guidelines and care programmes, as well as supporting efforts towards more knowledge-based, equal, and efficient care [43, p. 77]

In the last two excerpts above, from both reports, equality is assumed to be achieved through nationally defined guidelines, i.e. control over what knowledge is produced and used in a healthcare setting. It is not obvious at first sight how equality is attained through such knowledge production, but when considering the context of these statements, we understand equality as meaning providing the same care to all patients. Hence, knowledge production in terms of guidance and guidelines appears as a means of producing sameness; therefore, we argue that all five of the above articulations of equality produce a meaning of equality as reducing differences between the care given by different providers. This interpretation mirrors Höglund et al.’s [2] analysis of healthcare governance reports, where compliance with treatment and therapeutic guidelines was articulated as producing equality, and such equality was often represented as (geographical) sameness. This understanding of equality can thus be associated with the knowledge-governing approach of NPM [14].

### Equality as increased agency and responsibility for individual patients

In the HSC report, talk about *person-centred care* is mentioned rarely (only four times), and never articulated as related to equality, but in the PHC report, where person-centred care is a central term (mentioned 28 times), it is articulated as a way to produce equality, such as in the following:

Person-centred care is an important part of the transition to a modern and more equal health and medical care [36, p. 276]

The quote above does not articulate a particular meaning or explain how person-centred care will produce equality. However, in the quote below, referring to so-called patient contracts, it is explained that such contracts give healthcare providers:

better conditions for becoming more person-centred, which in itself is a means to achieve other goals, for example equality and efficiency [36, p. 222]

The patient contract reform is also described as follows:

The relationship between professional and patient becomes more equal when one has access to the same information about what is planned for me as a patient. It is also expected to contribute to increased equality between different patient groups, as the possibility of obtaining an individual plan, and thus a so-called patient contract, is expanded to reach more patients than today [36, p. 221]

In the two quotes above, a chain of equivalence is articulated as patient contract → person-centred care → equality between patient and professional *and* between different patient groups. Similar articulations are found elsewhere (36, p. 197; p. 221].

In contrast to the sections above on equality as sameness (sections 1a–c), here an individualised plan is highlighted as something that will increase equality between patients. An overall statement about what the reform in the PHC report is “all about” articulates person-centred care as:

A shift from seeing patients as recipients of care services to a shared partnership. Seeing patients as co-creators of their own health and care. This entails both a cultural and a power shift. This shift also creates the opportunity for those who wish and are able to take greater responsibility for their own care. Making this shift is also crucial for ensuring good and equal care for vulnerable and marginalised groups, as well as for those with the greatest need for health and medical services [36, p. 107]

The quote above articulates that the proposed shift, from seeing patients as mere recipients of care to partners and co-creators of their own health and care, will entail responsibilisation and a shift in power that it is crucial for “equal care for vulnerable and marginalised groups”. Thus, equality is articulated by way of allowing patients to take responsibility for their own health and care, which is particularly beneficial for the vulnerable and marginalised. A chain of equivalence is articulated as person-centred care → partnership → a shift in responsibility → the levelling of power → equal care. This establishes an understanding of equality as a matter of individual responsibility and equality occurring at an individual level, including among vulnerable and marginalised groups.

Such a “freedom-of-choice” discourse reveals some similarity to Burström’s [34] marketisation-oriented meaning of equality; “to maximise personal freedom and choice”. However, a patient choice orientation in healthcare has been problematised because it can be unfavourable for patients who lack adequate resources to make such beneficial choices [19, 20], in particular those “vulnerable and marginalised groups” for whom the PHC report promises to provide equality. We argue that Wedin’s [35] understanding of imaginary equality adds nuances to the label “marketisation discourse” of this meaning of equality. It is articulated as a normatively a-historical expectation that each individual subject should be granted recognition in accordance with their own self-understanding, without consideration of context. These policies are thus producers of a particular subject – vulnerable and marginalised yet responsible and empowered by de-contextualisation.

### Equality as being responsive to the diverse needs of different groups

The articulation of equality as providing care in accordance with needs is absent from the HSC report, but appears occasionally in the PHC report, such as:

The structures of the healthcare system need to be changed in order to better develop quality and accessibility and to promote efficiency and equality. Healthcare authorities need to become better at organising and delivering care in ways that respond to the differing needs and expectations of various target groups. Particular importance should be given to improving care for those with the greatest needs [36, p. 55]

This statement takes into account the fact that different groups might have different needs, thereby articulating egalitarian meanings of equality [34]. Sometimes the social aspects are also stated more explicitly:

In principle, everyone living in Sweden should have the same opportunity to access health and medical care when needed. The conditions for being able to turn to health and medical care services must, as far as possible, be equalised, since only care needs should determine the scope and nature of interventions. Other barriers preventing individuals from accessing care must be removed by society. Examples of such barriers include economic, social, and cultural barriers, as well as geographic ones [36, p. 276]

Here, equality is articulated as the equal opportunity to access health care and as determined by care needs. Social, economic, and cultural barriers are articulated as obstacles to be removed, not manifestations of unequal disparities in living conditions [34].

The following paragraph, however, explicitly highlights socio-economic groups:

Primary care is important in ensuring that healthcare is provided on equal terms and distributed equally across the population. It is widely recognised that healthcare today generally has difficulties reaching socioeconomically disadvantaged groups and meeting their health and medical care needs [36, p. 460]

Here, equality is articulated as access to care, and inequality as a system(atic) problem: a health system that fails to accommodate the needs of socio-economically “disadvantaged” groups. A strengthened and well-developed primary care service is framed as a solution to this:

Through clearer regulation of collaboration, primary care is strengthened, which in turn contributes to more equal health in the population. A well-developed primary care system improves access to care for the entire population. Groups with poorer socioeconomic conditions more often have more illnesses than other groups. As primary care focuses on the individual’s overall disease profile, it is expected to better meet the individual’s combined needs according to our proposals [36, p. 196].

These articulations, displaying similarities to Burström’s egalitarian equality [34], are rare across the material. The use of the terms “groups with poorer socioeconomic conditions” and “socioeconomically disadvantaged groups” produces subjects of lack or limitation. While it may seem to be admitting to structural inequality, the production of such subjects instead excludes structural thinking and draws attention away from the everyday practices of an unjust system through its focus on individuals and the constitution of certain groups. In addition, the last sentence in the above quote illustrates how the positioning of the problem shifts back to the individual level, to the individual clinical picture and their needs. In this way, structural inequalities are acknowledged but quickly translated into individualised needs to be managed by primary care.

## Concluding discussion

As illustrated in the analysis, we have identified different meanings of equal(ity) in the policies analysed; as sameness and uniformity (sections 1a, b, and c), increased agency and responsibility for individual patients (Sect.  2), and being responsive to care needs (Sect.  3, PHC report only). This illustrates that equality carries various and competing meanings. When a term has various meanings, they can shift depending on context, and become competing or simply unclear; thus, equality can be understood as a floating signifier. It floats between competing discourses that attempt to “fix” the meaning [40]. Teasing out floating signifiers can thus reveal locations of discursive contestation. Based on our empirical analysis, we conclude that “equality” *is* a term under contestation, chiefly between individualised care and responsibility on the one hand and, on the other, various aspects of geographical sameness, with a “sliver” of an egalitarian understanding. Burström’s [34] claim, that equality in care is a debate between the neoliberal maximisation of “personal freedom and choice” and the egalitarian mitigation of “disparities in human circumstances and opportunities” is not precisely what our analysis suggests. While our last section, on responsiveness to need, reveals some connections to an egalitarian understanding (especially in the PHC report), the different aspects of sameness do not fit well into a discourse of “personal freedom and choice”.

Considering instead the works of Wedin [35], and his historical analysis of Swedish education policies, we find that he identifies two traditional meanings of equality: the equal distribution of resources and the same treatment before the law. As Sect.  1 illustrates articulations of equality as sameness and uniformity, Wedin’s second meaning has some resonance with our results. But the articulations found in Sect.  2, on increased agency and responsibility for individual patients, connect better to Burström’s understanding of “personal freedom and choice”. This does not, however, capture the complexity we found in the meaning of agency and responsibility, but in fact aligns better with Wedin’s *imaginary* equality, which he argues to be dominant in modern Swedish education policies. An imaginary equality is articulated as a normative expectation that each individual subject should be addressed and granted recognition in accordance with their own self-understanding, almost like a blank slate. We consider this problematic because, rather than addressing the structural conditions that produce inequality, such an understanding of equality relocates the problem to the level of individual recognition [45]. While it may appear inclusive, it makes individual affirmation the measure of equality rather than the redistribution of resources or the dismantling of structural barriers [46]. In addition, by privileging the immediate present [47], this understanding of equality silences longitudinal thinking in matters of health equality.

The recurring use of categories such as “vulnerable and marginalised”, “groups with poorer socioeconomic conditions”, or “socioeconomically disadvantaged groups” produces subjects marked by lack and limitation. While these formulations ostensibly signal an awareness of inequality, through their formulation as static labels they simultaneously individualise and depoliticise it, excluding a structural and systemic processual understanding of inequality. In this sense, policy discourses do not merely describe existing realities but actively constitute a particular kind of subject, at once vulnerable and marginalised, yet imagined as empowered, responsible, and capable of self-management. This paradoxical production displaces attention from the social and institutional arrangements that generate inequality, transforming structural injustice into an issue of individual capacity and responsibility, thereby silencing the very conditions it purports to address.

Both the HSC report and the PHC report present equality as a central objective, and both articulate equality in terms of sameness and uniformity. However, they also articulate its meaning in different ways, with only the PHC report articulating equality in terms of being responsive to needs and also placing a lot more emphasis on individual agency and responsibility. We thus conclude that the dominant articulation of equality in contemporary Swedish healthcare policy is a combination of two “liberal” understandings, i.e. understandings that emphasise individual rights, autonomy, and equal opportunities. Firstly, equality as sameness: to receive the same treatment or quality of healthcare regardless of location and such care having the same availability regardless of place of residence. Secondly, recognition of patients’ agency and individual responsibility, meanings that arguably presuppose ignoring the context of which patients and caregivers are part. We have thus shown that these liberal and individualised understandings of equality are dominant, and that egalitarian understandings are excluded from the discourse of equality, in a process like what Lapidus [7, p. 13] calls rhetorical redescription, in which ideas about equal healthcare are reframed and reformulated. Previous research has shown that neoliberal reforms of the Swedish healthcare system have led to geographical service concentration and increased utilisation among affluent populations with lower healthcare needs [48, 49, 50].

Possibly, the meanings of equality found in these reports also reflect a shift in the meanings of equality over time, paralleling changes in other foundational welfare concepts. For instance, a striving for “security” has shifted from meaning material protection and the reduction of economic inequality, to freedom from (fear of) crime [51]; “freedom” from not being dominated by someone else, to individual choice [52]; and “freedom of choice” from providing citizens with the material stability that allows them to shape their own lives without fear of poverty or ill health, to the distribution of welfare services according to individual needs and preferences [53]. In this light, the dominant meanings of equality in healthcare that we have identified are not about reducing structural disparities, but about ensuring equal opportunities for uniform access and enabling individual choice and agency.

A notable pattern in the analysed policy texts is the frequent referral to regional variation and the relative ease with which geographical inequalities are articulated in both reports, compared to social inequalities related to class, race, or ethnicity. These articulations often frame geographical inequality as a logistical or administrative challenge, one that can be addressed through planning, coordination, and the redistribution of resources. While socio-economic conditions are occasionally mentioned in the PHC report, there is little discussion of how structural factors such as class, racialisation, migration status, or systemic discrimination shape access to care or health outcomes. One possible explanation is that geographical inequality aligns more easily with the administrative logic of the healthcare system, which is organised around regional governance. It is also arguably less politically sensitive, as it can be framed in neutral terms of distance or infrastructure. In contrast, articulating inequality in relation to class or ethnicity/race requires the acknowledgement of power relations, historical injustices, and systemic exclusion, which may challenge dominant liberal narratives of individual responsibility and equal opportunity.

The PHC report refers to equality in relation to the primary healthcare choice reform (without articulating a specific meaning of equality) and cites research showing that the choice reform has “favoured socioeconomically strong groups more than weak ones” [36, p. 299] (see also [48, 50]). Despite this acknowledgement, the reform does not propose any suspension of the obligatory choice model; instead, it highlights limitations in existing study designs and places responsibility on regions to “take equal care into account when designing healthcare choice models” [36, p. 300]. In effect, the obligatory choice model itself remains largely unquestioned (see also [49]).

Considering that the language available to us directs our way of thinking and acting, the exclusion of the egalitarian meaning of equality may impede our ability to envision a healthcare system that can take patients’, caregivers’, and the healthcare system’s context and the social structures connected to (ill) health into account. This discursive exclusion might be particularly problematic in a primary healthcare setting, which is often embedded in local communities and well positioned to work proactively with social determinants of health. Primary healthcare has the potential to act not only as a provider of medical services but also as a key institution for promoting social justice (see [54]), addressing structural inequities, and contributing to broader societal efforts to reduce health disparities (see also [34, 55]). Yet, when equality is narrowly framed as geographical sameness or individual responsibility, this transformative potential risks being overlooked or underutilised.

## Data Availability

The data analysed in the current study consist of publicly available government reports (Swedish Official Government Reports, SOU), which are cited in the reference list.

## Footnotes

1 All translations into English were made by us, after the analysis.
